# Determination of Rare Earth Elements by Inductively Coupled Plasma–Tandem Quadrupole Mass Spectrometry With Nitrous Oxide as the Reaction Gas

**DOI:** 10.3389/fchem.2022.912938

**Published:** 2022-06-29

**Authors:** Yanbei Zhu

**Affiliations:** National Metrology Institute of Japan (NMIJ), National Institute of Advanced Industrial Science and Technology (AIST), Tsukuba, Japan

**Keywords:** REEs, spectral interference, reaction cell, reaction gas, mass-shift, ICP-QMS/QMS

## Abstract

Nitrous oxide (N_2_O) was investigated as the reaction gas for the determination of rare earth elements (REEs) by inductively coupled plasma–tandem quadrupole mass spectrometry (ICP-QMS/QMS). The use of N_2_O as the reaction gas apparently improved the yields of ^*m*^M^16^O^+^ for Eu and Yb in the reaction cell. As a result, the sensitivities for measurement of Eu and Yb were apparently improved in comparison to those obtained with O_2_ as the reaction gas. A high sensitivity measurement of the whole set of REEs was achieved, providing a typical sensitivity of 300,000 CPS mL/ng for REEs measured with an isotope having isotopic abundance close to 100%. The use of N_2_O as the reaction gas helped suppress Ba-related spectral interferences with the measurement of Eu, permitting the measurement of Eu in a natural sample without mathematic correction of spectral interferences. The detection limits (unit, pg/mL) for 14 REEs (except for Pm) from La to Lu were 0.028, 0.018, 0.006, 0.026, 0.006, 0.010, 0.017, 0.006, 0.016, 0.010, 0.016, 0.004, 0.023, and 0.012, respectively. The validity of the present method was confirmed by determining REEs in river water-certified reference materials, namely, SLRS-3 and SLRS-4.

## Introduction

Rare earth elements (REEs) are a group of 17 transition metals, including Sc, Y, and 15 lanthanides, in the periodic table of chemical elements (Balaram, 2019). Nevertheless, the lanthanides are more often referred to as REEs in many research works, while Pm is usually not investigated due to the lack of a natural and stable isotope. In the present work, REEs stand for the lanthanides except for Pm.

Due to the specific physicochemical properties of REEs, they are usually studied as a group of tracers for geological, hydrological, environmental, and fossil fuel research (Zuma et al., 2020; Wysocka, 2021). Acknowledging the capabilities for multi-elemental analysis, lower detection limits at ng/kg or even pg/kg levels, and commercial availability in common chemical analysis laboratories, inductively coupled plasma mass spectrometry (ICP-MS) is becoming a dominant approach for quantitative analysis of REEs in various samples (Bandura et al., 2006).

The extremely high temperature of the argon plasma provides an ionization efficiency for all REEs close to 100%, permitting the high sensitivity measurements by ICP-MS. At the same time, polyatomic spectral interferences such as oxide and hydroxide are usually problematic for accurate quantitation of REEs in natural samples, for example, ^137^Ba^16^O^+^ and ^136^Ba^16^O^1^H^+^ interfering with the measurement of ^153^Eu^+^, and ^139^La^16^O^+^ interfering with the measurement of ^155^Gd^+^.

There are majorly three ways to solve the problem caused by spectral interferences. One is the mathematic correction based on the ratio of the signal intensity of an interfering polyatomic ion (for example, ^137^Ba^16^O^+^) to that of an interferent ion (for example, ^137^Ba^+^) (Seregina et al., 2018; Zhao et al., 2019; Carvalho et al., 2020; Kunzetsova et al., 2021). Mathematic correction is a straightforward way of canceling spectral interferences in the measurements by ICP-MS. However, the accuracy and the precision of an analytical result may be greatly deteriorated in case of a relatively large portion (for example, over 30%) of spectral interference being corrected due to the variance in the signal intensity of the interfering spectrum. The second one is suppressing the formation of an interfering polyatomic ion by removing an interferent element (for example, Ba) from REEs through chemical separation (Watanabe et al., 2018; Ito et al., 2019; Barrat et al., 2020; Bradley et al., 2020) or by using a desolvating device to remove water contents introduced into the argon plasma (to suppress the formation of solvent related ions, for example, O^+^, H^+^, and OH^+^) (Giardi et al., 2018; Gatiboni et al., 2020; Shen et al., 2022). The third one is separating an interfering polyatomic ion from an REE of interest by high-resolution ICP-MS (Giardi et al., 2018; Barrat et al., 2020; Bradley et al., 2020; Kiruba et al., 2021), collision/reaction cell ICP-MS (Gueguen et al., 2009; Arslan et al., 2018; Rojano et al., 2019; Trommetter et al., 2020; Baghaliannejad et al., 2021), and tandem quadrupole ICP-MS with a reaction cell (ICP-QMS/QMS) (Galusha et al., 2018; Zhang et al., 2019; Zhu, 2020; Zhu et al., 2021).

A previous work by the present author showed that ICP-QMS/QMS provides the best separation of interfering polyatomic ion from an REE of interest by measuring at a so-called mass-shift mode with oxygen as the reaction gas (Zhu, 2020). In ICP-QMS/QMS, an octopole reaction cell is sandwiched by using two quadrupole analyzers. For the measurement of REEs at the mass-shift mode, the parameters for the first quadrupole (QMS1) were set to permit the pass of an ion of interest, for example, mass to charge ratio (*m*/*z*) = 139 for ^139^La^+^ (along with ^138^Ba^1^H^+^), and to block ions with *m*/*z* ≠ 139. After entering the reaction cell, ^139^La^+^ reacted with O_2_ to form ^139^La^16^O^+^, while ^138^Ba^1^H^+^ did not form ^138^Ba^1^H^16^O^+^. These product ions were introduced to the second quadrupole (QMS2) whose parameters were set to permit the pass of an ion with *m*/*z* = 155. As a result, the ^139^La^16^O^+^ ions passed through and arrived at the detector, while the product ions of ^138^Ba^1^H^+^ were blocked by the second quadrupole.

The mass-shift mode by ICP-QMS/QMS with oxygen as the reaction gas was effective for separating spectral interferences in the measurement of REEs. However, since reactions for the formation of EuO^+^ and YbO^+^ are endothermic, the transference ratio from M^+^ to MO^+^ for Eu^+^ and Yb^+^ was significantly lower than that for other REEs, resulting in relatively lower sensitivities. Optimization of the octopole bias voltage helped to improve the formation of EuO^+^ and YbO^+^. Nevertheless, the transference ratio from M^+^ to MO^+^ for Eu^+^ and Yb^+^ was still apparently lower than that for other REEs (Zhu et al., 2021).

In addition to the commonly equipped reaction cell gases, namely, He, H_2_, O_2_, and NH_3_, N_2_O had been used in collision/reaction cell ICP-MS and ICP-QMS/QMS to improve the formation of oxide ions (Bandura et al., 2006; Harouaka et al., 2021). In the present work, N_2_O was investigated as the reaction cell gas for ICP-QMS/QMS to improve the formation of EuO^+^ and YbO^+^ to achieve the highest sensitivities for the measurement of all REEs. The optimized conditions were applied to the measurement of REEs in two natural water-certified reference materials, namely, SLRS-3 and SLRS-4.

## Materials and Methods

### Instruments

Measurements of REEs in the present work were carried out with an ICP-QMS/QMS instrument (Agilent 8800, Agilent Technologies Japan, Ltd.), for which the typical operating conditions are summarized in Table 1. These operating conditions were optimized to obtain the best performance for measuring REEs with the highest sensitivity. A Millipore purification system (Nihon Millipore Kogyo) was used to provide deionized water for the present experiment. A chemical balance (model XS205DU) purchased from METTLER TOLEDO was used for making samples and calibrating solutions, while the chemical balance was calibrated yearly by the Japan Calibration Service System (JCSS).

**TABLE 1:** Typical operating conditions of the ICP-QMS/QMS instrument.

Parameter	Value	Unit
RF power	1,550	W
Sampling depth	8.0	mm
Plasma gas flow rate	14.0	L min^−1^
Carrier gas flow rate	0.80	L min^−1^
Makeup gas flow rate	0.50	L min^−1^
Extraction 1 lens	−6.0	V
Extraction 2 lens	−220	V
Omega bias lens	−165	V
Omega lens	22.2	V
Cell gas flow rate	30	%
ORC inlet	−100	V
ORC outlet	−70	V
Octopole bias	(−5, −10, −15, −20, and −25)*^a^*	V
Deflecting lens	(3.0, −2.4, −7.8, −13.6, and −15.0)*^a^*	V
Energy discrimination	−7.0	V
Analytical mode	Mass-shift	-
Integration time	1.0	s
Number of replicates	10	-

a Deflecting lens was optimized to match each value of the octopole bias.

### Chemicals and Samples

The following chemicals were purchased from Kanto Chemical Co., Inc.: single-element standard solutions (1,000 mg/L) of REEs and barium; Ultrapur^®^ grade HNO_3_ (60% in mass). Two river water CRMs (SLRS-3 and SLRS-4) were purchased from the National Research Council of Canada and analyzed to confirm the validity of the present method. The CRM samples were acidified to 0.3 mol/L of nitric acid by adding concentrated nitric acid, where this dilution factor was calculated and applied to the results to obtain the initial concentrations of REEs. Calibrating solutions for measurement by ICP-QMS/QMS were also prepared in 0.3 mol/L of nitric acid. Standard N_2_O gas (99.5%, 8 kg/m^2^, 5 L) was purchased from AS ONE Corp. and used as the reaction cell gas for ICP-QMS/QMS.

### Calibrating Method

The concentrations of REEs in the present work were obtained based on a standard addition method (Zhu et al., 2018). Spiked and non-spiked sub-samples were made for each sample and subjected to the measurement by ICP-QMS/QMS. The natural content of yttrium in the sample was used as the internal standard. The concentrations of REEs in a spiked sub-sample for standard addition were over two folds of those in the initial sample to ensure the precision of calibration.

## Results and Discussion

### Dependence of Relative Signal Intensities of La, Eu, and Yb on the Octopole Bias of ICP-QMS/QMS

Measurement of REEs by ICP-QMS/QMS at the mass-shift mode (^*m*^M^+^ → ^*m*^M^16^O^+^) with O_2_ as the reaction gas was effective for separating spectral interferences. However, the production ratios of EuO^+^ and YbO^+^ were significantly lower than those of other REEs because the reactions for the formation of EuO^+^ and YbO^+^ were endothermic, while those for other REEs were exothermic. A relatively stronger negative voltage applied to the octopole bias resulted in limited improvement of signal intensities for the measurement of Eu and Yb (Zhu, 2021). In the present work, N_2_O was used as the reaction gas for ICP-QMS/QMS to improve the production ratios of oxide ions of Eu^+^ and Yb^+^, while La^+^ was investigated as a representative of other REEs.

**FIGURE 1:**
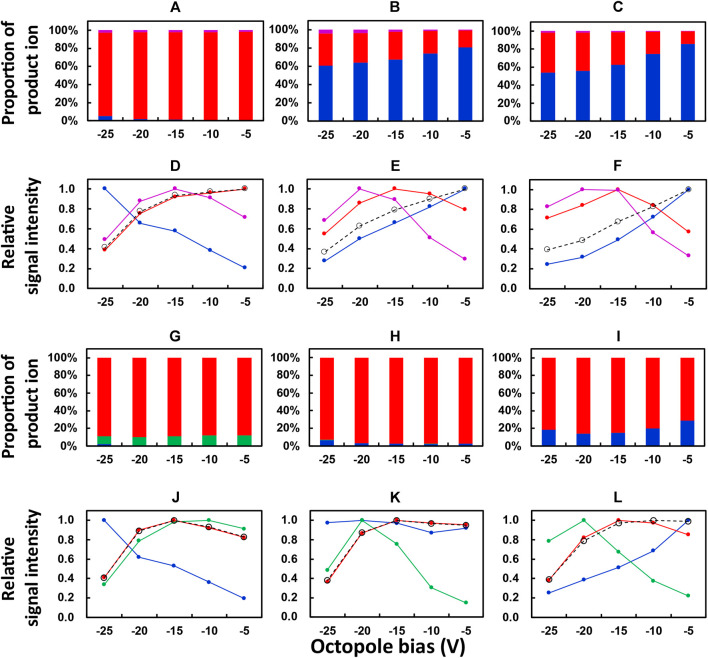
Dependence of the proportion of product ions and relative signal intensities on the octopole bias of ICP-QMS/QMS. Reaction gas: O_2_ for **(A–F)**; N_2_O for G to L. Isotope monitored: ^139^La^+^ for **(A,D,G,J)**; ^153^Eu^+^ for **(B,E,H,K)**; ^172^Yb^+^ for **(C,F,I,L)**. Color of the plot: blue, ^*m*^M^+^; red, ^*m*^M^16^O^+^; purple, ^*m*^M^16^O_2_^+^; green, ^*m*^M^14^N^+^; black, the sum of (^*m*^M^+^, ^*m*^M^16^O^+^, and ^*m*^M^16^O_2_^+^) or (^*m*^M^+^, ^*m*^M^16^O^+^, and ^*m*^M^14^N^+^). Reproducibility of the data, relative standard deviation under 2 %; *n* = 10.

The dependence of signal intensities of La, Eu, and Yb on the octopole bias of ICP-QMS/QMS was investigated with O_2_ and N_2_O, respectively, as the reaction gases. The results are plotted in Figure 1. The default value of the octopole bias (suggested for mass-shift measurement with O_2_ as the reaction gas) was −5 V, and the range for octopole bias investigated in the present work was from −25 V to −5 V with a footstep of 5 V.

A preliminary test in the present work was performed to check the major product ions from an REE ion ^*m*^M^+^ when O_2_ and N_2_O were, respectively, used as the reaction gases. The results showed that the major product ions were ^*m*^M^+^, ^*m*^M^16^O^+^, and ^*m*^M^16^O_2_^+^ and ^*m*^M^+^, ^*m*^M^16^O^+^, and ^*m*^M^14^N^+^, respectively. Therefore, a study on the dependence of relative signal intensities on octopole bias was focused on these ions. Figures 1A–C and Figures 1G–I showed the proportion of product ions (for example, ^139^La^+^, ^139^La^16^O^+^, ^139^La^16^O_2_^+^) in the sum of those obtained from one precursor ion (for example, ^139^La^+^). Figures 1D–F and Figures 1J–L showed the relative signal intensity, which was calculated as the relative value of the signal intensity for an ion in comparison to its maximum value in the range of the octopole bias investigated. The results for Figures 1A to 1F and 1G to 1L were, respectively, obtained with O_2_ and N_2_O as the reaction gases.

As can be seen from Figures 1A–C, the dominating product ions from ^139^La^+^, ^153^Eu^+^, and ^172^Yb^+^ obtained at an octopole bias of −5 V were ^139^La^16^O^+^, ^153^Eu^+^, and ^172^Yb^+^, respectively. This can be attributed to the exothermic producing reaction of ^139^La^16^O^+^ and the endothermic ones for ^153^Eu^16^O^+^ and ^172^Yb^16^O^+^, when O_2_ was used as the reaction gas. When the octopole bias was changed to a stronger negative voltage until −25 V, the proportions of ^153^Eu^16^O^+^ and ^172^Yb^16^O^+^ increased from around 20% to around 40%, while that of ^139^La^16^O^+^ was not varied apparently. These results indicated that a stronger negative voltage of the octopole bias improved the relative yields of ^*m*^M^16^O^+^ ions whose producing reactions are endothermic, which can be attributed to the increase of the velocity and the resulting collision energy of the precursor ion entering the reaction cell. However, such an increase in the velocity also results in a shorter retention time in the reaction cell and a lower reaction rate of an exothermic reaction. This contributed to the decrease in the relative signal intensity of ^139^La^16^O^+^ and the increase in that of ^139^La^+^ when the octopole bias changed from −5 V to a stronger negative voltage until −25 V, as shown in Figure 1D. By contrast, the maximum values of relative signal intensities of ^153^Eu^16^O^+^ and ^172^Yb^16^O^+^ (Figures 1E,F) were obtained at an octopole bias of −15 V, which can be attributed to the increased yields of these ions due to the elevated collision energy in comparison to the octopole bias of −5 V. Moreover, the decrease in the sum of relative signal intensities of product ions (black plots, Figures 1D–F) may indicate the decrease in transmittance of the ions when a stronger negative voltage was applied to the octopole bias. Based on these results, an octopole bias of −15 V was selected as the compromised optimum condition for measuring the whole set of REEs at relatively higher sensitivities.

The results obtained with N_2_O as the reaction gas are plotted in Figures 1G–L in a similar way to those in Figures 1A–F for the results obtained with O_2_. It is notable that ^*m*^M^14^N^+^ ions are plotted in Figures 1G–L instead of ^*m*^M^16^O_2_^+^ ions plotted in Figures 1A–F.

It can be seen from Figure 1G that the major product ion from ^139^La^+^ was ^139^La^16^O^+^ when N_2_O was used as the reaction gas, with a portion comparable to that obtained with O_2_ as the reaction gas (Figure 1A). By contrast, instead of the dominating production ions as ^153^Eu^+^ (Figure 1B) and ^172^Yb^+^ (Figure 1C) obtained with O_2_ as the reaction gas, ^153^Eu^16^O^+^ (Figure 1H) and ^172^Yb^16^O^+^ (Figure 1I) accounted for the dominating portions when N_2_O was used as the reaction gas. This can be attributed to the decrease in reaction energy for producing ^153^Eu^16^O^+^ and ^172^Yb^16^O^+^ when N_2_O was used instead of O_2_ (Harouaka et al., 2021).

As can be seen in Figures 1J–L, the highest relative signal intensities of ^139^La^16^O^+^, ^153^Eu^16^O^+^, and ^172^Yb^16^O^+^ were observed at an octopole bias of −15 V, which was selected as the optimum condition for measuring the whole set of REEs with N_2_O as the reaction gas.

### Comparison of Normalized Sensitivities for the Measurement of REEs by ICP-QMS/QMS

The sensitivity for measuring an element by ICP-QMS/QMS depends on the sample uptake rate, the ionization rate, the abundance of the isotope selected for measurement, the transmittance in the ion lens system, and the yield of the product ion in the reaction cell. In the case of measuring REEs, the parameters for all REEs are quite similar, except for the abundance of the isotope and the yield of the product ion.

Therefore, normalized sensitivities of REEs were obtained to cancel the effect of the abundance of the isotope and to elucidate the contribution of the improved yield of the product ion by using N_2_O as the reaction gas. A normalized sensitivity (*S* ^*^) was calculated from the initial sensitivity (*S* ^0^) and the abundance of an isotope (*A*) based on Eq. 1. If the yields of product ions of interest were close to 100% for all REEs, the normalized sensitivities are expected to be close to one another.

S∗=S0A. (1)

**FIGURE 2:**
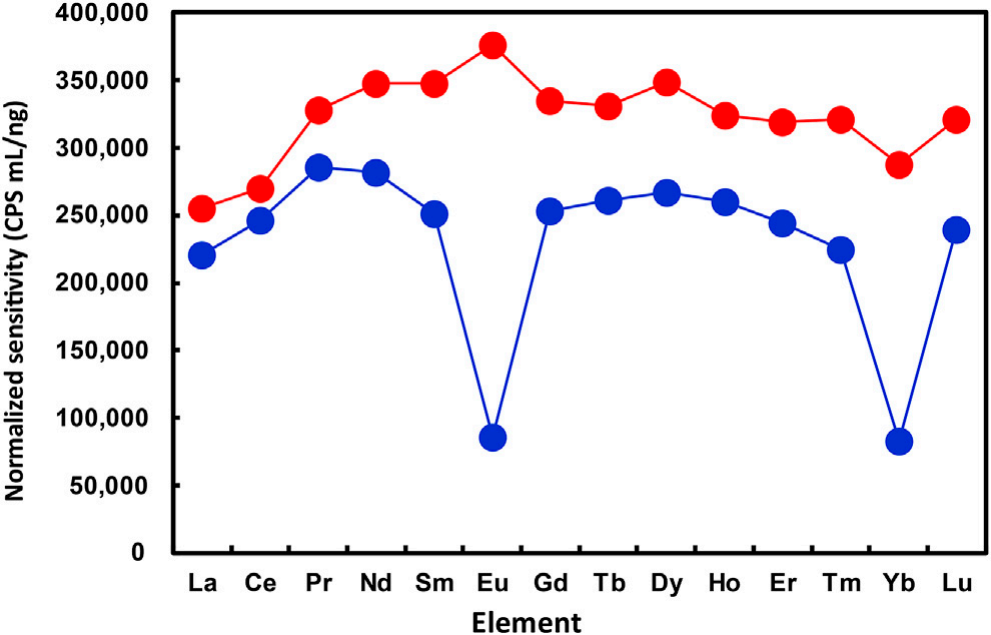
Comparison of normalized sensitivities for REEs obtained at different operating conditions of ICP-QMS/QMS. Reaction gas: O_2_ for blue plots; N_2_O for red plots. Reproducibility of the data, relative standard deviation under 2 %; *n* = 10.

The normalized sensitivities for REEs obtained in the present work are plotted in Figure 2, with O_2_ and N_2_O, respectively, used as the reaction gases. The octopole bias was set to -15 V for both O_2_ and N_2_O. As can be seen from Figure 2, the normalized sensitivities for all REEs were generally at a similar level when N_2_O was used as the reaction gas. The relatively lower normalized sensitivities of La and Ce might be attributed to the formation of ^*m*^M^14^N^+^ ions (over 10% of the product ions), as indicated in Figure 1G.

By contrast, the normalized sensitivities of Eu and Yb were apparently lower than those of other REEs, when O_2_ was used as the reaction gas. Moreover, the relative sensitivities for all REEs obtained with N_2_O as the reaction gas were higher than those with O_2_ as the reaction gas. These results indicated that the optimized operating condition with N_2_O as the reaction gas provided higher yields of ^*m*^M^16^O^+^ ions for all REEs and helped achieve higher sensitivities for measuring the whole set of REEs by ICP-QMS/QMS.

### Ba-Related Spectral Interferences With the Measurement of Eu by ICP-QMS/QMS

Due to relatively smaller differences (one to two orders of magnitudes) among the concentrations of REEs in a natural sample, spectral interferences of oxide ions of lighter REEs (with a smaller atomic number) with the measurement of heavier REEs (with a larger atomic number) can be effectively suppressed by measuring at the mass-shift mode of ICP-QMS/QMS with O_2_ as the reaction gas. However, the concentrations of Ba in natural samples are usually much higher (for example, over four orders of magnitudes) than those of Eu, whose both stable isotopes (^151^Eu and ^153^Eu) suffer from spectral interferences from oxides and hydroxides of Ba. As a result, mathematical correction of Ba-related spectral interferences is still required even for the measurement by ICP-QMS/QMS with O_2_ as the reaction gas (Zhu and Itoh, 2021).

**FIGURE 3:**
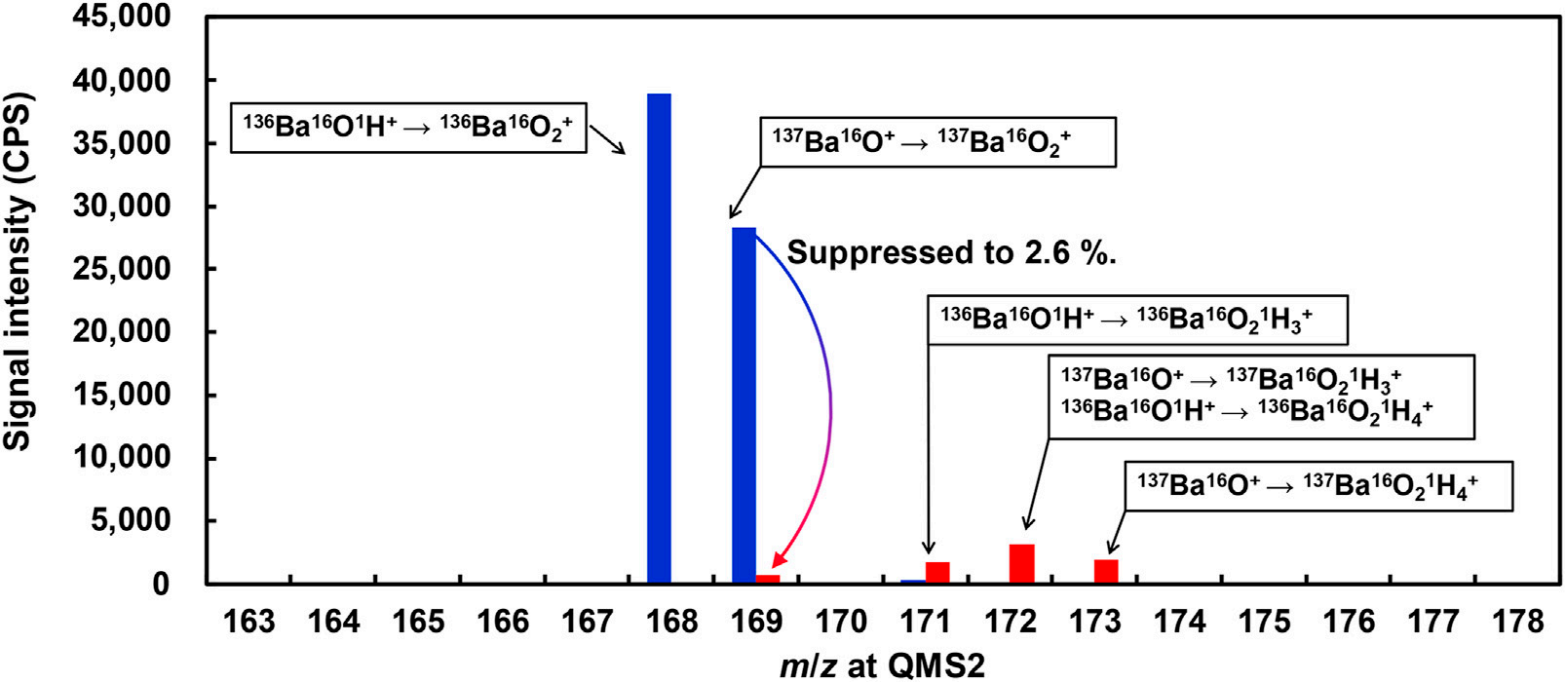
Comparison of Ba-related spectral interferences on the measurement of ^153^Eu^+^ by ICP-QMS/QMS. Reaction gas: O_2_ for blue plots; N_2_O for red plots. Octopole bias: −15 V. Seeing for QMS1, m/z = 153. Reproducibility of the data, relative standard deviation under 2 %; *n* = 10

Ba-related spectral interferences with the measurement of ^153^Eu^+^ were further investigated in the present work, with O_2_ and N_2_O as the reaction gases, respectively. The investigation was carried out by introducing 10 mg/L Ba standard solution to the ICP-QMS/QMS and measuring the product ions of ^153^M^+^ (*m*/*z* = 153 for QMS1) at the *m*/*z* range of 160–180 for QMS2, covering the spectra of ^153^Eu^16^O^+^. The results are plotted in Figure 3.

As can be seen from Figure 3, the major product ions obtained with O_2_ as the reaction gas were ^168^M^+^ and ^169^M^+^, which can be attributed to ^136^Ba^16^O_2_^+^ and ^137^Ba^16^O_2_^+^, respectively. By contrast, the major product ions obtained with N_2_O as the reaction gas were ^169^M^+^, ^171^M^+^, and ^172^M^+^ and ^173^M^+^, which can be attributed to ^137^Ba^16^O_2_^+^, ^136^Ba^16^O_2_H_3_^+^ (^137^Ba^16^O_2_H_3_^+^ + ^136^Ba^16^O_2_H_4_^+^), and ^137^Ba^16^O_2_H_4_^+^, respectively. The source of hydrogen for product ions obtained with N_2_O as the reaction gas might be attributed to gaseous impurities.

It is notable that the signal intensity of ^137^Ba^16^O_2_^+^ was suppressed to 2.6% by using N_2_O as the reaction gas instead of O_2_, indicating much less spectral interferences with the measurement of ^153^Eu^16^O^+^.

### Analytical Figures of Merits for Measurement of REEs by ICP-QMS/QMS

Sensitivity, blank equivalent concentration (BEC), and detection limit (DL) are usually provided as analytical figures of merits for the measurement of the elements by ICP-MS. These parameters are evaluated and summarized in Table 2, respectively, with O_2_ and N_2_O as the reaction gases.

**TABLE 2:** Comparison of sensitivity, BEC, and DL values for REEs measured by ICP-QMS/QMS with O_2_ and N_2_O, respectively, as the reaction gases (octopole bias, -15 V).

Element	*m*/*z*	O_2_			N_2_O		
		Sensitivity (CPS mL/ng)	BEC (pg/mL)	DL (pg/mL)	Sensitivity (CPS mL/ng)	BEC (pg/mL)	DL (pg/mL)
La	139	220,985	0.008	0.030	254,917	0.008	0.028
Ce	140	217,929	0.005	0.024	238,483	0.007	0.018
Pr	141	285,257	0.003	0.017	327,937	0.001	0.006
Nd	146	48,482	0.003	0.022	59,753	0.007	0.026
Sm	147	37,733	0.006	0.043	52,160	0.002	0.006
Eu	153	44,838	0.004	0.024	**196,360**	**0.002**	**0.010**
Gd	157	39,670	0.002	0.011	52,342	0.003	0.017
Tb	159	261,392	0.002	0.007	331,227	0.001	0.006
Dy	163	66,508	0.005	0.033	86,904	0.002	0.016
Ho	165	260,269	0.002	0.010	324,374	0.002	0.010
Er	166	81,849	0.003	0.020	106,869	0.004	0.016
Tm	169	224,416	0.002	0.012	320,798	0.001	0.004
Yb	172	18,069	0.009	0.060	**62,655**	**0.006**	**0.023**
Lu	175	233,119	0.004	0.019	312,566	0.004	0.012

The sensitivity was obtained as the signal intensity corresponding to each of 1.0 ng/mL REEs. A BEC value was obtained as the concentration equivalent to the average of five measurements of 0.3 mol/L HNO_3_ solution, where each measurement contains 10 repetitions (1 s/repetition) for each REE. A DL value was obtained as the concentration corresponding to 3-fold of the maximum standard deviation obtained in five measurements of the 0.3 mol/L HNO_3_ solution.

As can be seen from Table 2, the sensitivity, BEC, and DL values for each REE obtained with N_2_O as the reaction gas were generally superior to those obtained with O_2_, especially for Eu and Yb due to the improved yields of ^*m*^M^16^O^+^ ions in the reaction cell. It is notable that the sensitivities summarized in Table 2 were the initial values without normalization to the isotopic abundances. As a result, the sensitivity depended on the isotopic abundance of an isotope measured. When N_2_O was used as the reaction gas and an REE was measured at an isotope with an isotopic abundance of 100%, the typical sensitivity was around 300,000 CPS mL/ng. The BEC and DL values for each REE with N_2_O as the reaction gas were, respectively, lower than 0.01 pg/mL and 0.03 pg/mL, sufficiently low for the direct measurement of REEs in most natural water samples. The data for Eu and Yb with N2O as the reaction gas are shown in bold fonts in Table 2 to highlight the improved analytical performance.

### Results for the Recovery Test of Spiked REEs in River Water CRMs

Recovery tests of spiked REEs in river water CRMs were carried out by adding a mixed solution of REEs into river water CRMs, SLRS-3 and SLRS-4, respectively. The concentration of each REE in these spiked samples was elevated by 200 pg/mL. The concentrations of REEs in these spiked samples were determined along with those in the non-spiked SLRS-3 and SLRS-4 samples. A recovery value (*R*) was calculated based on Eq. 2, where, $C_{spiked}^{o}$ and $C_{non-spiked}^{o}$ were the observed values of an REE in the spiked sample and the non-spiked sample, and $C_{spike}^{c}$ was the spiked concentration.

R=(Cspikedo−Cnon−spikedo)Cspikec. (2)

The results of recovery tests are summarized in Table 3. It can be seen from Table 3 that each recovery value is close to 100%, indicating that the concentrations of REEs spiked to the river water CRMs can be accurately determined by ICP-QMS/QMS with O_2_ or N_2_O as the reaction gas. It is notable that the standard deviation values for La, Ce, Pr, Tb, Ho, Tm, and Lu were around 1.0%, which can be attributed to the excellent sensitivities due to the isotopes measured having high isotopic abundance. The standard deviation values for other REEs were generally in the range of 1–2%, except for those of Yb (roughly 3%) measured with O_2_ as the reaction gas. Such relatively lower standard deviation values of Yb were improved to a level similar to those of other REEs when N_2_O was used as the reaction gas, attributable to the improved yields of ^172^Yb^16^O^+^ in the reaction cell.

**TABLE 3:** Recoveries of spiked REEs in river water CRMs measured by ICP-QMS/QMS with O_2_ and N_2_O, respectively, as the reaction gases.

Element		Recovery (%)^a^														
		O_2_ as the reaction gas								N_2_O as the reaction gas						
		SLRS-3				SLRS-4				SLRS-3				SLRS-4		
La		98.0	±	0.7		98.2	±	0.7		101.1	±	0.7		100.1	±	0.7
Ce		98.5	±	0.6		98.1	±	0.7		100.9	±	0.6		101.2	±	0.5
Pr		100.0	±	1.2		100.6	±	0.8		100.9	±	1.0		98.9	±	0.9
Nd		98.5	±	1.4		98.1	±	1.9		100.1	±	1.4		97.8	±	1.2
Sm		98.2	±	1.7		98.4	±	2.0		99.0	±	1.7		99.4	±	1.3
Eu		99.7	±	1.9		100.5	±	2.0		100.5	±	0.8		98.3	±	0.7
Gd		100.5	±	2.0		100.4	±	1.6		101.3	±	2.1		98.4	±	1.1
Tb		99.3	±	1.1		99.4	±	0.6		100.6	±	0.6		98.2	±	0.8
Dy		100.3	±	1.2		99.0	±	1.0		99.7	±	2.0		99.4	±	0.8
Ho		100.1	±	0.7		99.9	±	0.8		100.4	±	0.7		99.4	±	1.0
Er		100.0	±	1.5		99.7	±	1.0		100.7	±	1.6		99.4	±	1.0
Tm		99.2	±	0.9		99.7	±	1.2		100.3	±	1.0		98.6	±	0.6
Yb		99.2	±	2.7		100.2	±	2.9		100.7	±	1.2		99.0	±	1.7
Lu		99.6	±	0.9		100.3	±	0.9		100.4	±	1.3		99.2	±	1.2

a Recovery value is shown as (mean ± standard deviation, *n* = 10).

### Analytical Results of REEs in River Water CRMs

The concentrations of REEs in two river water CRMs, namely, SLRS-3 and SLRS-4, were determined in the present work. The results are summarized in Table 4 along with reported data.

**TABLE 4:** Analytical results of REEs in river water CRMs (Unit, pg/mL).

Element		SLRS-3												
		This work (O_2_)				This work (N_2_O)				Brenner et al. (1998)				Halicz et al. (1999)
La		239	±	4		233	±	2		210	±	1		250
Ce		265	±	3		264	±	3		250	±	1		293
Pr		57.2	±	1.2		56.2	±	1.2		53.0	±	0.5		61
Nd		228	±	7		225	±	6		200	±	2		239
Sm		43.8	±	2.5		44.0	±	1.5		39.0	±	1.5		43
Eu		6.62	±	0.39		6.68	±	0.47		6.6	±	0.5		6.6
Gd		29.7	±	1.3		28.4	±	1.1		28.0	±	1.6		39
Tb		3.75	±	0.22		3.68	±	0.17		3.6	±	0.1		4.5
Dy		19.9	±	1.0		19.9	±	0.7		19.8	±	0.6		22
Ho		3.82	±	0.13		3.88	±	0.16		3.8	±	0.1		4.9
Er		11.4	±	0.7		11.5	±	0.3		11.0	±	0.3		14
Tm		1.62	±	0.09		1.55	±	0.11		1.5	±	0.1		1.6
Yb		10.6	±	1.4		10.3	±	0.7		9.4	±	0.2		12
Lu		1.53	±	0.16		1.64	±	0.14		1.4	±	0.1		1.6
**Element**		**SLRS-4**												
		**This work (O**_**2**_ **)**				**This work (N**_**2**_ **O)**				**Compiled (Zhu et al., 2021)**				
La		297	±	4		282	±	3		291	±	9		
Ce		360	±	5		351	±	4		363	±	9		
Pr		69.2	±	1.2		68.9	±	1.1		71.1	±	2.4		
Nd		275	±	6		268	±	3		271	±	6		
Sm		60.0	±	2.0		57.7	±	1.6		57.6	±	1.8		
Eu		7.88	±	0.62		7.74	±	0.21		8.44	±	0.57		
Gd		33.7	±	1.4		32.3	±	1.5		34.2	±	1.8		
Tb		4.36	±	0.22		4.27	±	0.22		4.32	±	0.14		
Dy		23.2	±	0.9		21.9	±	0.9		23.6	±	1.0		
Ho		4.37	±	0.19		4.24	±	0.05		4.66	±	0.27		
Er		12.6	±	1.1		12.6	±	0.7		13.2	±	0.8		
Tm		1.79	±	0.13		1.70	±	0.13		1.82	±	0.08		
Yb		12.0	±	0.6		11.9	±	0.8		12.2	±	0.7		
Lu		1.80	±	0.13		1.72	±	0.07		1.91	±	0.10		

It can be seen from Table 4 that the results for each CRM obtained with O_2_ and N_2_O, respectively, as the reaction gas agreed with each other considering the standard deviation of each value. However, it is notable that the measurement of Eu with O_2_ as the reaction gas suffered approximately 10% of Ba-related spectral interference, which was mathematically corrected. By contrast, Ba-related spectral interference with the measurement of Eu was negligible when N_2_O was used as the reaction gas and did not require mathematic correction. The concentrations of Ba in both samples were approximately 15 ng/mL, roughly four orders of magnitude higher than the concentration of Eu.

The present results for REEs in SLRS-3 were generally close to those reported by Brenner et al. (1998), who applied mathematic corrections to the measurements of La, Ce, and Eu regarding Ba-related spectral interferences. The DLs for REEs achieved by Brenner et al. (1998) were in the range from 0.02 pg/mL for Sm to 0.2 pg/mL for Nd, roughly 10-fold higher than those obtained in the present work. By contrast, the results by Halicz et al. (1999) showed slightly higher values for most REEs except for Sm, Eu, Tm, and Lu, perhaps indicating some spectral interferences, even mathematic correction applied to the measurements of ^151^Eu^+^ regarding the spectral interference by ^135^Ba^16^O^+^. The DLs for REEs obtained by Halicz et al. (1999) were in the range from 0.005 pg/mL for Tm to 0.1 pg/mL for Nd. Brenner et al. (1998) and Halicz et al. (1999), respectively, used a Meinhard type pneumatic concentric nebulizer and an ultrasonic nebulization system to introduce the samples to their ICP-MS instruments. The present results for REEs in SLRS-4 obtained in the present work agreed with the compiled data based on multiple works (Zhu et al., 2021).

These results for REEs in SLRS-3 and SLRS-4 showed that the present method is effective for the determination of REEs in river water samples.

## Conclusion

The application of N_2_O as the reaction gas for ICP-QMS/QMS provided excellent analytical figures of merits for the determination of REEs. In comparison to O_2_ that was usually used as the reaction gas, N_2_O improved the yields of ^*m*^M^16^O^+^ ions for Eu and Yb in the reaction cell and permitted measuring them at much higher sensitivities.

The application of N_2_O as the reaction gas also contributed to the suppression of Ba-related spectral interferences with ^153^Eu^+^ to 2.6% of that observed with O_2_ as the reaction gas. This merit permitted the measurement of Eu without mathematic correction even with Ba concentrations over four orders of magnitude higher.

The analytical results for spiked REEs in SLRS-3 and SLRS-4 provided recovery values quite close to 100%. The concentrations of REEs in SLRS-3 and SLRS-4 determined by the present method were incidence with those reported, indicating the validity of the method.

## Data Availability

The original contributions presented in the study are included in the article, further inquiries can be directed to the corresponding author.
